# Fertility preservation in Hong Kong Chinese society: awareness, knowledge and acceptance

**DOI:** 10.1186/s12905-020-00953-3

**Published:** 2020-04-29

**Authors:** Suet Ying YEUNG, Elaine Yee Lee Ng, Terence Tzu Hsi LAO, Tin Chiu Li, Jacqueline Pui Wah CHUNG

**Affiliations:** Department of Obstetrics and Gynaecology, The Chinese University of Hong Kong, Prince of Wales Hospital, 1/F, Block E, Special Block, Prince of Wales Hospital, Shatin, N.T Hong Kong SAR

**Keywords:** Fertility preservation, Hong Kong, Surveys and questionnaires

## Abstract

**Background:**

Fertility preservation for both medical and non-medical reasons are gaining increasing attention world-wide. This study aimed to evaluate the awareness, knowledge and acceptance on fertility preservation in Hong Kong Chinese.

**Methods:**

This was a cross-sectional study carried out between June 2016 to March 2017. A self-administered questionnaire was distributed by convenience sampling.

**Results:**

Forty two percent of subjects returned the questionnaire (*n* = 296/697). Among them, only 54.3% were aware of any means of fertility preservation. Tertiary educated individuals are more aware than less educated individuals (73.6% versus 46.6%, *p* < 0.001). The most commonly known method is oocyte cryopreservation (94.3%). Most of the respondents (80%) were interested to know about fertility preservation, 84.1% considered fertility preservation counselling necessary and 83.3% would consider undergoing fertility preservation if a treatment has a high chance of causing infertility despite the possibility of delay in cancer treatment. Up to 93.9% agreed to set up a dedicated referral centre with government funding and 73.4% agreed that fertility preservation for medical indication should be provided as a government funded service. In terms of fertility preservation for non-medical reason, 65.5 and 70.4% agreed that fertility preservation should be offered to single men and women without partner respectively, while only 53.3 and 50% agreed that fertility preservation is acceptable for delay in childbearing for career development in female and male respectively.

**Conclusion:**

There was a low awareness but positive attitude towards fertility preservation among Hong Kong Chinese. Acceptance towards medically indicated fertility preservation is high while a considerable number also accepts it for non-medical reasons. Educational campaigns are required to arouse awareness of fertility preservation to prevent individuals being deprived of the option due to lack of knowledge. Dedicated referral centre with established efficient patient referral pathways and financial support should be provided to improve the provision and uptake of fertility preservation service to enhance the reproductive potential and life options of women.

## Background

Fertility preservation is an emerging field in reproductive medicine. The advancement of reproductive technology has made childbearing possible in patients suffering from diseases or treatments that are potentially detrimental to fertility as well as healthy individuals who want to postpone their family planning for various social reasons.

The majority of fertility preservation for medical indication is due to gonadotoxic treatments for cancer and autoimmune conditions, as well as other benign conditions that may impair fertility. Cancer survival has improved globally over the past decades [1, 2] and many patients survive till reproductive age. Preserving fertility is an important aspect of quality of life of survivors [3]. Multiple international guidelines have stated the need for fertility counselling before gonadotoxic treatment for cancer survivors [4, 5] yet many patients were not made aware of their options [6]. Multiple physician factors have been reported including the lack of awareness and knowledge, perceived priority of cancer treatment to fertility need, and difficulty in initiating the discussion in the time of emotional crisis [7]. Health-care policy issues such as a lack of government funding or health insurance coverage, and service gaps hindering timely referral to specialist service also prohibit patients from seeking fertility preservation [8].

Elective oocyte cryopreservation for non-medical purposes has become increasingly popular in both Western communities and developing world [9, 10]. An increase in trend towards delaying childbearing has been observed in women of reproductive age due to various lifestyle factors, such as career development and absence of partner. A recently published study showed that the acceptance to elective oocyte freezing is as high as 89% in UK and Denmark [9]. This has given rise to a lot of social and ethical debate worldwide and the gaining popularity of fertility preservation for non-medical reason has significant implication on policy making and resources allocation in public health [11, 12].

Despite being one of the most developed part of the world, fertility preservation service is largely underutilized and of limited availability in Hong Kong [13]. Previous local survey has revealed an unexpectedly low awareness of fertility preservation among Hong Kong clinicians [14]. It has also been reported that Hong Kong young adults has a low awareness of fertility decline and a lower motivation to seek fertility solution compared to their western counterparts [15]. This might be explained by the influence of traditional Chinese culture which led to avoidance of the discussion of fertility issues because of a fear of social stigma. Most of the research on fertility preservation awareness were conducted in Western countries and there was no published report in the Chinese population. We therefore conducted this survey to explore the awareness, knowledge and acceptance of Chinese regarding fertility preservation. This information will allow appropriate allocation of resources to establish a culturally and administratively acceptable public education program and building up of a dedicated referral system to improve provision of fertility preserving service.

## Methods

This was a cross-sectional survey to evaluate the awareness, knowledge and acceptance on fertility preservation among Hong Kong Chinese public. The study was conducted between June 2016 to March 2017. Ethical approval for the study was obtained from the institutional Survey and Behavioral Research Ethics Committee (SBREC).

The instrument of the study was a self-administered questionnaire with a brief introduction to explain about the survey. The questionnaire was specifically developed for this study and consisted of a total of 25 items in two parts. The first part included questions on the baseline demographics, education and religious background of the participant. The second part included questions on the awareness, attitude and knowledge on fertility preservation. Their views on the demand of this service, the factors that they would consider in making any decisions for fertility preservation were examined. Practical questions on the potential costs and the need for a dedicated fertility preservation clinic were ascertained. The questionnaire was distributed to a pilot group of 30 before it derived at its final version for this study. Participants were invited to comment on the content, clarity and length of the questionnaire before the study questionnaire was finalized.

The study questionnaires were randomly distributed to any individuals accessible through the public area of Obstetrics and Gynaecology, Urology and Surgical out-patient clinics at Prince of Wales Hospital, Hong Kong between June 2016 to March 2017. Medical staffs and medical students were excluded from the study. A short oral introduction of the study was made, and subjects were made aware that the questionnaire was voluntary. If the subjects agreed to participate, the questionnaire, together with a returning envelope were given to them and they were asked to mail back the questionnaire after completion anonymously.

SPSS for Window package (SPSS Inc. Chicago, Illinois Version 20) was used for data entry and analysis. Demographic data was summarised with the use of means, medians and percentages. The Chi-square test (χ2 test) was used for categorical data and Student’s t test (t test) for continuous variables. Results with *p*-value of < 0.05 were statistically significant.

## Results

Six hundred and ninety-seven questionnaires were distributed within the study period. A total of 296 questionnaires were completed and returned to the investigators, giving a response rate of 42.5%. Table 1 summarizes the baseline demographics of the respondents. Some of the respondents did not answer all questions; hence the denominators of each response are stated.

**Table 1:** Socioeconomic and demographic characteristics of respondents

Characteristics	Respondents (***n*** = 296)
**Gender**	
Male	56 (18.9)
Female	166 (56.1)
Not specified	74 (25)
**Marital status**	
Single	107 (36.1)
Married/cohabiting	174 (58.8)
Divorced/separated	7 (2.4)
Widowed	1 (0.3)
Not specified	7 (2.4)
**Religion**	
No	185 (62.5)
Protestants	60 (20.3)
Catholic	15 (5.1)
Buddhism	21 (7.1)
Hinduism	2 (0.7)
Others	1 (0.3)
Not specified	12 (4.1)
**Age**	
< 18	4 (1.4)
18–34	136 (45.9)
35–43	94 (31.8)
> 44	54 (18.2)
Not specified	8 (2.7)
**Education level**	
No formal education	2 (0.7)
Primary level	4 (1.4)
Secondary level	99 (33.4)
Postsecondary	41 (13.9)
Tertiary level	142 (48)
Not specified	8 (2.7)
**Occupation**	
Full-time job	232 (78.4)
Part-time job	19 (6.4)
Housewife	12 (4.1)
Retired	4 (1.4)
Unemployed	9 (3)
Students	14 (4.7)
Not specified	6 (2)
**Monthly domestic household income**^**a**^	
Low income	64 (21.6)
Middle income	149 (50.3)
High income	73 (24.7)
Not specified	10 (3.4)

^a^Low income: less than USD 2500; middle income: USD 2500 to 6400; high income: more than USD 6400 (Classification based on Hong Kong Census and Statistic Department Household income distribution report 2016)

### Awareness and acceptance of fertility preservation and source of information

Two hundred and twenty-five respondents (i.e. 77.8%, *n* = 225/289) were aware that treatment for some diseases can have an adverse effect on fertility while only 54.3% (*n* = 157/289) of respondents were aware of any means of fertility preservation. Among them, the most commonly known strategy was oocyte cryopreservation (95.5%, *n* = 150/157), followed by sperm cryopreservation (94.3%, *n* = 148/157) and embryo cryopreservation (58.0%, *n* = 91/157). Significantly fewer respondents were aware of fertility sparing surgery, radiation shielding, ovarian or testicular cryopreservation (ranged from 12.1 to 18.4%, *n* = 19–29/157). Majority of the respondents were made aware of fertility preservation by information from mass media and news (*n* = 101/157, 64.3%), newspaper (*n* = 62/157, 39.4%) and less from their healthcare providers (*n* = 58/157, 36.9%). The awareness of fertility preservation was no different among different gender, age group nor marital status, yet people who had received tertiary education are more likely to be aware of fertility preservation compared to those less educated (73.6% versus 46.6%, *P* < 0.001).

Only 21.7% (*n* = 63/286) of the respondents knew where to seek fertility preservation advice or service. A minority of respondents (10.0%, *n* = 29/289) were aware of the existing legislations or regulations of fertility preservation service in Hong Kong.

### Attitude towards fertility preservation and factors for consideration of fertility preservation

Majority of respondents considered fertility preservation counselling necessary if a treatment has a moderate or high chance of causing infertility (75.3 and 84.1%, *n* = 214/284 and *n* = 239/284). Similarly, most would consider undergoing fertility preservation if there is a moderate or high chance of infertility following treatment (71.6 and 83.3%, *n* = 202/284 and 235/284).

**Fig. 1:**
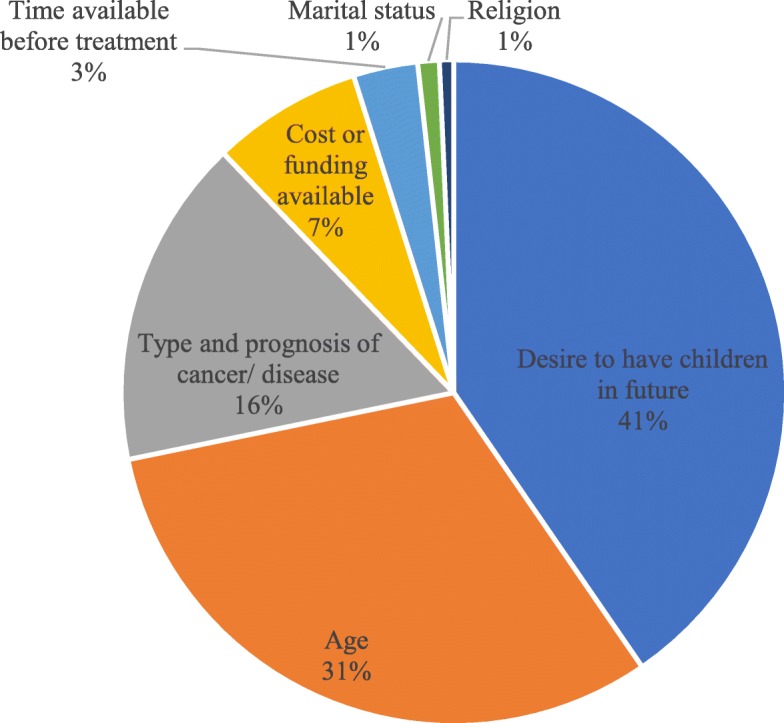
Single most important factor to consider when deciding fertility preservation (*n* = 287)

The single most important factor they would consider to decide whether to proceed to fertility preservation is shown by Fig. 1. More than half of the respondents still wish to receive counselling (58.3%, *n* = 165/283) and attempt for fertility preservation procedures (60.2%, *n* = 170/282) despite that this might cause treatment delay of up to 4 weeks.

More than 90% of all respondents accepts fertility preservation service for medical indications including cancer, exposure to occupational hazards that might affect fertility and men with poor sperm quality. Whereas for non-medical reasons such as single women without partner or delaying childbearing for career development, the acceptance decreases to 50 to 70% (Table 2).

**Table 2:** Attitude on fertility preservation for different reasons

Fertility preservation should be provided for	No. of respondents (%)
A. Medical indication	
Delay in family planning due to cancer or cancer treatment (*n* = 287)	274 (95.4%)
Individuals who have exposure to occupational hazard (radiation/ chemical exposure) (*n* = 270)	270 (93.8%)
Men with poor semen quality wants to save sperms for future use (*n* = 288)	263 (91.3%)
B. Non-medical reasons	
Delay in family planning due to career development in females (*n* = 287)	153 (53.3%)
Delay in family planning due to career development in males (*n* = 286)	143 (50.0%)
Single women who wants to freeze their eggs before finding their partner (*n* = 287)	202 (70.4%)
Single men who wants to freeze their sperm before finding their partner (*n* = 287)	188 (65.5%)
Couple who wants to have embryos frozen for future use when it is time for second child (*n* = 283)	202 (71.3%)

### Funding and education

Nearly all of the respondents agreed that setting up a dedicated centre for fertility preservation is necessary and that funding should be provided by the government for this purpose (93.9%, *n* = 266/283 and 92.0%, *n* = 267/290 respectively). Most respondents (75.4%, *n* = 218/289) agreed that fertility preservation procedures for medical indication should be provided as a government funded service but less than a quarter agreed that the service should be fully subsidized.

Up to 80.0% (*n* = 224/280) of respondents were interested to know more about fertility preservation. Most of the respondents (95.5%, *n* = 277/290) considered educational materials important for information gathering and decision-making in patients undergoing fertility preservation counselling, in the form of social media or websites (83.0%, *n* = 240/289), symposia or talks (74.0%, *n* = 214/289) and educational pamphlets (65.4%, *n* = 189/289).

## Discussion

In the past, cancer treatment has been focused on improvement of survival as the utmost priority while quality of life of the survivors was often disregarded. Nevertheless, cancer patients of childbearing age considered the potential loss of fertility as painful as confronting cancer itself [16] and that they were most concerned with the impact of cancer treatment on their fertility [17]. Fertility preservation has become an important part of cancer management program with great impact on quality of life [3]. Benign conditions such as ovarian endometriomas, major thalassemia and autoimmune diseases may also adversely affect the ovarian reserve and leading to loss of fertility potential. In recent years, elective fertility preservation for non-medical reasons has gained more public attention due to delay in child bearing for various lifestyle or social reasons and increasing awareness of age-related fertility decline. This has led to increasing debates on the benefits and risks, as well as the ethical implication of fertility preservation for non-medical indication.

In Hong Kong, the currently established fertility preservation service included sperm cryopreservation, mature oocyte cryopreservation and embryo cryopreservation. Gonadal tissue cryopreservation is still limited to research settings. Access to fertility preservation service is limited as these services are only provided in two University facilities and a few private reproductive medicine centres. No public or government funded fertility preservation service is available for all indications including cancer patients. There are no established referral pathways or designated fertility preservation centres for fertility preservation counselling and only a minority of cancer patients received information about fertility preservation service and underwent the procedure. The Council on Human Reproductive Technology of Hong Kong only provides guidelines of and regulates gamete or embryo handling and storage, while there is no local fertility preservation society to promote the awareness and monitor the usage of fertility preservation service.

Our study is the first to evaluate the awareness of and attitude to fertility preservation in the Chinese community. Our findings provide information on the major barriers against fertility preservation and offered insight to strategies to remove these barriers and guide the development of fertility preservation service and research in Chinese locality.

We found a low awareness of fertility preservation among the respondents with only half of them ever heard of the concept of fertility preservation and only minority of them knew where to seek the service. Surveys in the western society has showed a much higher degree of awareness with up to 90% of reproductive age women aware of means of fertility preservation [18–20]. This might be explained by a lack of public education as well as the cultural tradition that fertility issues were considered sensitive and seldom publicly discussed. Nevertheless, there was a positive attitude with high acceptance towards fertility preservation and respondents were eager to explore more on this issue. This indicated an imperative need for action to provide public education to prevent depriving individuals from desiring fertility preservation services due to lack of knowledge. Most respondents reported that their source of information was traditional media. In recent years, social media platforms such as Facebook, Instagram or Twitter has gained popularity in the younger generation and we should fully utilize these platforms in addition to traditional means for health education. The awareness is particularly low in low educated participants; this highlights the need of specific strategies to target the less well-educated population whom rely on traditional media such as TV programs or newspaper as their major access to health information. Public advertisement or promotion programs at mass media level, territory-wide public campaigns and advertising activities at the community level (such as health talks) are essential to reach this group of population. Interestingly, only 36.9% of the participants obtained their information about fertility preservation from their healthcare providers. This finding echoes our former report of a low awareness of fertility preservation among Hong Kong physicians [14]. A number of strategies can target at raising medical professionals’ awareness and knowledge. Examples included introducing fertility preservation as part of the medical curriculum at undergraduate level; organizing seminars, workshops, and conferences regularly and actively invite physicians and nurses especially those specialties involved in managing patients who potentially require this service to participate. Information about fertility preservation service referral pathways could be distributed to them to encourage active referral for patients in need.

Financial aspect is crucial in decision making for fertility preservation [8]. In Hong Kong, sperm cryopreservation costs around USD1500–1650, while oocyte and embryo cryopreservation procedures are even more expensive (USD15000–20000). Unlike many of the western communities where fertility preservation procedures are partially or fully funded by government or health insurance, the cost of these procedures and the subsequent storage were entirely self-financed in Hong Kong. Many could only see these as a luxury they cannot afford, as some of the cancer treatments might already exhausted their savings. Government funding can allow more patients to be able to afford such expensive endeavors. Majority of our respondents agreed that preserving procedures should be provided as a government funded service, although less than a quarter agreed that the costs should be completely covered. Most of our respondents agreed that a dedicated centre for referral should be set up, and similar response was obtained from our former study on the opinion of local clinicians [14]. This highlighted a consensus demand of an efficient patient referral pathway with easy public access to fertility preservation facilities.

Our former study targeting medical practitioners in Hong Kong revealed that more than half of the clinicians expressed the concerns of potential delays in cancer treatment for fertility preservation procedures, which impeded their discussion with patients, with up to 23% considering this as the single most significant factor when deciding on fertility preservation [14]. Surprisingly, only 3% of respondents considered time delay to treatment as the single most important factor when considering fertility preservation. Most were willing to postpone treatment for up to 4 weeks for this purpose. This highlighted the discrepancy in the perceived importance of preserving fertility potential between clinicians and patients. In fact, the introduction of random-start ovarian stimulation protocols nowadays allows oocyte retrieval to be achieved in a much shorter time interval compared to the past. Clinicians therefore should not hesitate to explore their patient’s desire for fertility preservation and early referral to a qualified specialist is crucial to avoid unnecessary delay in starting the procedure, and in turn the subsequent treatment. Appropriate fertility preservation procedures should be a joint decision between the oncologist, fertility specialist and the patient based on the individual clinical situation and time available before life-saving treatment without causing significant delay.

Nearly all respondents considered fertility preservation procedures acceptable for medical reasons but more cautious for non-medical reasons. Only around half of the respondents considered delay in family planning for career development an acceptable reason for fertility preservation in women. Similar studies in the western communities revealed a much higher awareness and acceptance to elective oocyte cryopreservation. An online cross-sectional survey conducted in Denmark and UK with 973 women reported that 89% considered elective oocyte cryopreservation acceptable and up to 19% expressed active interest in the procedure [9]. Another survey in Ireland reported similar findings with 89.7% women were aware of oocyte cryopreservation and 72.2% would consider freezing their eggs to preserve fertility in future [18]. Well-known companies in the west such as Facebook™ or Apple™ are already providing benefits to their employees to cover costs of fertility preservation. Our population are much more conservative towards fertility preservation for non-medical indication and this could be attributed to the fact that fertility issues remained a sensitive topic in traditional Chinese culture and that fewer open discussions for example, by celebrities are observed in our local society.

Our study has several limitations. This study was designed as a preliminary study with a limited sample size. Participants were randomly recruited by convenience sampling at hospital outpatient clinic of a single university. The aim is to reach a large population in a limited time to expedite data collection and maximize the number of recruitments but the subjects may not be representative for the entire population. Moreover, respondents who were willing to participate in this survey may represent a self-selected group with more interest in fertility preservation which is unavoidable in this form of study. This limited the generalizability of the results.

In addition, our current survey was unable to reveal the reasons behind the attitude uncovered and future studies with larger sample size are required to address this issue. Nonetheless, this study was the first to evaluate the view on fertility preservation from public’s perspective in Hong Kong Chinese population. It provides important insights to the attitudes and demand of fertility preservation in a Chinese population. This information is valuable in planning of fertility preservation service and educational campaigns to promote awareness and knowledge of fertility preservation in patients and general public in Hong Kong.

## Conclusions

Our study showed an overall positive attitude towards fertility preservation among Hong Kong Chinese and highlighted the expectation of general public in fertility preservation information and service. Acceptance to medically indicated fertility preservation is high. While more than half of the individuals also accept fertility preservation for non-medical reasons. Educational campaigns utilizing all mass media and social media are required to raise patient and public awareness of fertility preservation in Hong Kong. Dedicated referral centre needs to be set up to establish efficient patient referral pathways and financial support should be provided by the government to improve the uptake of fertility preservation service.

## Supplementary information

**Additional file 1.** Fertility Preservation Questionnaire.

## Data Availability

The datasets used and/or analysed during the current study are available from the corresponding author on reasonable request.
