# Supplementary material for: Fertility preservation in Hong Kong Chinese society: awareness, knowledge and acceptance
Source: BMC Womens Health. 2020 Apr 29;20:86. doi: 10.1186/s12905-020-00953-3 (PMC7189503; doi:10.1186/s12905-020-00953-3)
Supplement: Supplementary file 1 — Additional file 1. Fertility Preservation Questionnaire. [file 12905_2020_953_MOESM1_ESM.pdf]

Fertility Preservation Questionnaire

The ovaries and testes are the vital organs in the human reproductive system which is most sensitive to injury from medications, diseases, chemotherapy and radiation. Individuals can be exposed to these agents from treatment of cancers and other medical conditions such as autoimmune disease like systemic lupus erythematosus and haematological diseases, with subsequent gonadal damage and infertility. Those who survived the treatment and are planning to raise or complete their family are faced with the problems of gonadal injury and possible infertility in the background of advancing age. If their fertility can be preserved before such treatment is performed, especially at a younger age, then they would have the option to choose to regain their fertility after treatment, and empowered to choose whether to raise a family or not.

If damage to reproductive organs from treatment is likely, freezing gametes, embryos or gonadal tissue may help to preserve fertility or hormonal function. Fertility preservation refers to strategies aimed at preserving one's fertility as well as hormonal function against the damage of gonadotoxic treatment that could render the patient infertile.

We are now conducting a survey to assess the patient's awareness, behavior and knowledge on fertility preservation. The information will be of great value in setting up a fertility preservation service in Hong Kong in the near future. We hope that you can generously spare 5-10 minutes to complete the questionnaire. If you decide to participate in this study after reading and fully understood the relevant information given to you, please complete the questionnaire and mail it back to us in the attached self-addressed stamped envelope. Your participation is totally voluntary. All data collected are for the sole purpose of this research and is treated as highly confidential. You have the right to withdraw from the study at any time. If you have any queries concerning this survey or on fertility preservation, please feel free to contact the undersigned. Thank you very much!

Yours sincerely,

Dr. Chung Pui Wah, Jacqueline, Assistant Professor

Prof. Li Tin Chiu , Professor

Department of Obstetrics and Gynaecology

The Chinese University of Hong Kong, Prince of Wales Hospital

Tel: 26321537

Email: jacquelinechung@cuhk.edu.hk

Website: http://www.ivfhk.com.hk

**Part 1 Socio-demographics**	
**Age**	1. <18<br>2. 18-34<br>3. 35-43<br>4. ≥44
**Sex**	1. Female 2. Male
**Marital status**	1. Single<br>2. Married/cohabiting<br>3. Divorced/separated<br>4. Widowed
**Number of children**	1. Not applicable<br>2. Yes, Number: ________ Children
**Education**	1. No formal education<br>2. Primary level<br>3. Secondary level<br>4. Postsecondary<br>5.Tertiary level or above
**Occupation**	1. Student<br>2. Full-time job<br>3. Part-time job<br>4. Housewife<br>5. Retired<br>6. Unemployed<br>7. Others: _________(please specify)
**Are you taking government allowance?**	1. Yes 2. No
**Total family income per month**	HKD: _________
**Religion**	1. Buddhism<br>2. Catholic<br>3. Christian<br>4. Hinduism<br>5. Others: ______(please specify)<br>6. No

Part 2: Awareness about fertility preservation

1. Do you know that treatment for some diseases can have an adverse effect on fertility?
   a. Yes
   b. No
2. Have you heard of fertility preservation before?
   a. Yes → Go to Q3.
   b. No → Go to Q5.
3. Which type of fertility preservation have you heard of?
   (Can indicate more than one)
   a. Fertility-sparing surgeries
   b. Radiation shielding
   c. Sperm freezing
   d. Egg freezing
   e. Embryo freezing
   f. Ovarian tissue or testicular tissue freezing
4. How did you come across fertility preservation?
   a. Undergone it myself
   b. From relatives/friends
   c. From doctors/nurses
   d. From media/news
   e. From news paper
   f. From magazines
   g. From website
   h. Others: ____________________________________(please specify)

5. Have ***you yourself*** undergone any form of fertility preservation before?
   a. Yes
   b. No

6. Do you want your doctor to discuss the options of fertility preservation to you if your treatment for your disease has (Please answer every sub-questions) :

   a. a high chance of causing infertility? a. Yes b. No
   b. a moderate chance of causing infertility? a. Yes b. No
   c. minimal chance of causing infertility? a. Yes b. No

7. Will you consider fertility preservation if the treatment for your disease is known to have (Please answer every sub-questions) :
   a. a high chance of causing infertility? a. Yes b. No
   b. a moderate chance of causing infertility? a. Yes b. No
   c. minimal chance of causing infertility? a. Yes b. No

8. What are the factors do you think you will consider when deciding for fertility preservation? (can choose more than one)

   a. Age
   b. Time available before start of gonadotoxic treatment
   c. Type of cancer
   d. Prognosis of cancer or medical condition
   e. Resources available
   f. Desire to have children
   g. Marital status
   h. Cost
   i. Religion
   j. Others: ________________(please specify)

9. What is the SINGLE MOST IMPORTANT factor do you think you will consider when deciding for fertility preservation? (Please choose ONE only)
   a. Age
   b. Time available before start of gonadotoxic treatment
   c. Type of cancer
   d. Prognosis of cancer or medical condition
   e. Resources available
   f. Desire to have children
   g. Marital status
   h. Cost
   i. Religion
   j. Others: _______________(please specify)

10. Would you still want to be referred to see a fertility specialist to discuss options of fertility preservation if this may cause delay in your treatment by <u>(Please answer every sub-questions)</u> :

a. 1 week?	a. Yes	b. No
b. 2 weeks?	a. Yes	b. No
c. 4 weeks?	a. Yes	b. No
d. 8 weeks?	a. Yes	b. No
e. 12 weeks or longer?	a. Yes	b. No

11. Would you still want to attempt fertility preservation if this may cause delay in your treatment by <u>(Please answer every sub-questions)</u> :

a. 1 week?	a. Yes	b. No
b. 2 weeks?	a. Yes	b. No
c. 4 weeks?	a. Yes	b. No
d. 8 weeks?	a. Yes	b. No
e. 12 weeks or longer?	a. Yes	b. No

12. Do you know where to see doctors for fertility preservation?

a. Yes
b. No

13. Do you think setting up a dedicated clinic/centre for fertility preservation is necessary in Hong Kong?
    a. Yes
    b. No

14. What do you think is a reasonable price range for patients to pay for the ***initial consultation and assessment*** about fertility preservation (HKD)?

    a. <$500
    b. $500-999
    c. $1,000-1,999
    d. ≥$ 2,000
    e. It should be provided freely or funded by the government

15. What do you think is a reasonable price range for patients to pay for the ***initial workup*** (e.g. blood taking for ovarian reserve testing, semen analysis) about fertility preservation (HKD)?

    a. <$500
    b. $500-999
    c. $1,000-1,999
    d. ≥$ 2,000
    e. It should be provided freely or funded by the government

16. What do you think is a reasonable price range for patients to pay for **the *assisted reproductive technology*** (e.g. intrauterine insemination, in-vitro fertilization +/- intracytoplasmic insemination) for fertility preservation (HKD)?

    a. <$10,000
    b. $10,000-49,999
    c. ≥$50,000
    d. It should be provided freely or funded by the government

17. What do you think is a reasonable price range for patients to pay for ***the storage of frozen eggs/sperm/gonadal tissue per year*** for fertility preservation (HKD)?

    a. <$1,000
    b. $1,000-4,999
    c. ≥$5,000
    d. It should be provided freely or funded by the government

18. Do you think fertility preservation should be provided as a public service?
    a. Yes

b. No

19. Do you think the government should provide funding to set up a clinic/centre for fertility preservation?
    a. Yes
    b. No

20. Do you think that educational materials are important for fertility preservation counselling to enhance patient information gathering and decision making?
    a. Yes
    b. No

21. What kind of materials do you think is useful when counselling patients for fertility preservation? (can choose more than one)
    a. Education pamphlets
    b. Website
    c. Talks/lectures/ symposiums
    d. Videos
    e. Others: ________________(please specify)
    f. Not required

22. Have you heard of legislations/regulations regarding fertility preservation?
    a. Yes
    b. No

23. Do you think we should set any age limit on fertility preservation?
    a. Yes. What age do you think is appropriate?
        (1) upper limit:
            (a) no limit
            (b) up to age 45
            (c) up to age 35
            (d) up to 30
        (2) lower limit:
            (a) no limit
            (b) after puberty
            (c) above 18
            (d) above 25
            (e) above 30
    b. We should not set any limit at all

24. Do you agree that fertility preservation should be provided for the following reason?

Delayed family planning due to treatment of cancer	Yes	No
Delayed family planning due to career development in females	Yes	No
Delayed family planning due to career development in males	Yes	No
Single women who wants to freeze their eggs when they are young before they find their other half	Yes	No
Single men who wants to freeze their sperm when they are young before they find their other half	Yes	No
Individuals who may have exposure to occupational hazards like radiation/ chemical exposure	Yes	No
Men with poor semen quality wants to have sperm frozen for future use	Yes	No
Couple who wants to have embryos frozen for future use when it is time for second child	Yes	No

25. Do you want to know more about fertility preservation?
   a. Yes
   b. No

Thank you!
